# The SARS-CoV-2 ORF10 is not essential *in vitro* or *in vivo* in humans

**DOI:** 10.1371/journal.ppat.1008959

**Published:** 2020-12-10

**Authors:** Katarzyna Pancer, Aleksandra Milewska, Katarzyna Owczarek, Agnieszka Dabrowska, Michał Kowalski, Paweł P. Łabaj, Wojciech Branicki, Marek Sanak, Krzysztof Pyrc

**Affiliations:** 1 Department of Virology, National Institute of Public Health-National Institute of Hygiene; Warsaw, Poland; 2 Małopolska Centre of Biotechnology; Jagiellonian University; Kraków, Poland, Europe; 3 Microbiology Department, Faculty of Biochemistry, Biophysics and Biotechnology, Jagiellonian University; Krakow, Poland; 4 Department of Internal Medicine, Faculty of Medicine, Jagiellonian University Medical College; Kraków, Poland; Erasmus Medical Center, NETHERLANDS

## Abstract

SARS-CoV-2 genome annotation revealed the presence of 10 open reading frames (ORFs), of which the last one (ORF10) is positioned downstream of the N gene. It is a hypothetical gene, which was speculated to encode a 38 aa protein. This hypothetical protein does not share sequence similarity with any other known protein and cannot be associated with a function. While the role of this ORF10 was proposed, there is growing evidence showing that the ORF10 is not a coding region. Here, we identified SARS-CoV-2 variants in which the ORF10 gene was prematurely terminated. The disease was not attenuated, and the transmissibility between humans was maintained. Also, *in vitro*, the strains replicated similarly to the related viruses with the intact ORF10. Altogether, based on clinical observation and laboratory analyses, it appears that the ORF10 protein is not essential in humans. This observation further proves that the ORF10 should not be treated as the protein-coding gene, and the genome annotations should be amended.

## Author summary

Coronaviral genomes code for several proteins, with the large 1a/1ab being expressed directly from genomic (g)RNA. For the expression of other viral proteins, a set of subgenomic mRNAs is produced during replication. It includes mRNAs for structural (S-E-M-N) and accessory proteins. While the function of structural proteins is well described, the function of the latter ones is under debate. Some of them are required for replication, while others are dispensable in vitro but essential in vivo. Initially, 10 open reading frames (ORFs) were annotated in the SARS-CoV-2 genome, amongst which ORF10 is the most peculiar, as it does not share sequence homology with any known protein. Shortly after the genomic sequences became available, speculations on this protein's role in pathogenesis and innate immunity breaching started. Here, we identified two patients infected with SARS-CoV-2 variants with the ORF10 gene prematurely terminated. The disease was not attenuated, and the transmissibility was maintained. The in vitro study showed that the ORF10 is also not essential for replication. Consequently, ORF10 should not be treated as the protein-coding gene, and the genome annotations should be amended.

## Introduction

Coronaviruses are mammalian and avian RNA viruses, with large genomes of ~30,000 bases, which encode several proteins required for the virus replication, modulating the immune responses, and forming the scaffold of progeny virions [1]. The spatial distribution of the open reading frames (ORFs) is similar across the taxa. The 1a/1ab ORF starts near the 5’ terminus and is the only ORF that may be translated directly from the genomic RNA, giving rise to the non-structural proteins that re-shape the cellular microenvironment and initiate the replication process. Downstream, a number of ORFs encoding the structural proteins are located (HE, S, M, E, N), interspaced with genes encoding accessory proteins, varying in number and position [1]. SARS-CoV-2 genome annotation revealed 10 ORFs, of which the last one (ORF10) is positioned downstream of the N gene [2]. It is a hypothetical, 117 nt—long ORF, which was speculated to encode a 38 aa protein [2,3]. Bioinformatic analyses revealed that this hypothetical protein does not share the sequence similarity with any other known protein, and the predicted structure cannot be associated with a function. Nonetheless, it was speculated that the ORF10 protein may play a role in the immunogenicity of the SARS-CoV-2 or may modulate the virulence of the SARS-CoV-2. On the other hand, there is growing evidence showing that the ORF10 is not a coding region. Jungreis *et al*. analyzed the region for different *Sarbecoviruses* and found that only in a minority of cases, for the closest SARS-CoV-2 relatives, the ORF10 is intact. The evidence for the presence of the subgenomic mRNAs corresponding to the ORF10 is limited [4,5].

Here, we identified two patients infected with the SARS-CoV-2 virus, in which the ORF10 gene was prematurely terminated with a stop codon. The disease was not attenuated, and the transmissibility was maintained. Isolation of these viruses in cell culture showed that also *in vitro*, these strains replicated similarly to the related viruses with the intact ORF10. Altogether, based on clinical observation and laboratory analyses, it appears that the ORF10 protein is not essential for replication in humans.

## Results and discussion

The first SARS-CoV-2 infected patient was identified in Poland on 4^th^ March 2020, and since then, the genetic drift of the virus was monitored. The phylogenetic analysis led to the conclusion that the diversity of the virus is similar to the one observed worldwide [6]. The virus was introduced to the population of Poland from different sources, as hallmarks of different clades are present; virtually all genetic clades identified thus far were present [6]. In the course of analysis, some isolates showed some peculiarities. In two samples, sequencing revealed the disruption of the ORF10, as a stop codon was present at position aa 29. This premature termination results from the C-T mutation, amending the CAA to TAA codon, what is characteristic for coronaviral genomes [7,8]. The sequence of this particular region was covered >260 times, and no minority variants were detected. Interestingly, our submission of the sequence to the GenBank database was rejected due to the stop codon in the ORF10 gene. Further, *in silico* analysis of the genome data available revealed that taking into account the ORF size, the number of samples with mutations resulting in premature termination was noticeably higher in ORF10 than in other ORFs, with the ORF8 being an exception. This indicates reduced selection pressure on these two ORFs. Notably, the result obtained for ORF8 is in line with a recent report of Pereira *et al*., who suggested that a functional ORF8 protein is not necessary for SARS-CoV-2 persistence [9]. The coefficients obtained for ORF10, ORF8, and other ORFs, are shown in Table 1.

**Table 1:** The frequency of prematurely terminated sequences per ORF.

ORF	number of prematurely terminated sequences	Gene length	Coefficient of occurrence
S	28	3822	0.052
ORF3a	37	828	0.317
E	4	228	0.125
M	4	669	0.042
ORF6	51	186	1.948
ORF7a	102	366	1.98
ORF7b	30	132	1.615
ORF8	187	366	**3.63**
N	6	1260	0.034
ORF10	57	117	**3.461**

As we already knew that both original samples carried this mutation, we analyzed the accessible clinical data. A 58-year-old Polish man living in Warsaw, Poland, spent a few days in Germany at the end of February 2020. After returning to Poland, he was informed that he was in contact with the person infected with the SARS-CoV-2 virus. Despite the lack of apparent symptoms, he contacted a public health center. On the 4^th^ day from the exposure (the 5^th^ March 2020), a throat swab was collected and transported in a saline medium. The same day, a real-time RT-PCR (RT-qPCR) analysis was carried out in the National Institute of Public Health–NIH, in Warsaw using the Primerdesign, genesig Real-Time PCR CoVID-19 kit. RT-qPCR, according to Charite protocol, was used for verification of the result [10]. The sample was stored and sequenced (hCoV-19/Poland/PL_P32/2020). A symptomatic infection developed in due course and the patient experienced fatigue and loss of smell and taste. Respiratory symptoms were not reported. The infection lasted for 26 days, and the person recovered with no sequelae. On 6^th^ March 2020, the patient’s wife (female, 62 years) was examined, and the result was inconclusive. However, the second sample collected on 13^th^ March 2020 was positive for the SARS-CoV-2 RNA. The sample was stored and sequenced (hCoV-19/Poland/PL_P33/2020). The patient experienced fever (38°C) for 2 days and recovered without sequelae. Further, the sample was also collected from another person that was in contact with the first case (male, 41 years). The sample was tested positive for the SARS-CoV-2 RNA. No sample was collected for sequencing. The patient experienced a dry cough and loss of smell and taste. The infection lasted for 21 days, and the person recovered with no sequelae.

Based on the collected data, one may safely assume that the virus with the disrupted ORF10 was infectious and pathogenic in humans. The identical change in two patients proves that it did not result from intra-patient genetic drift and that the virus transmissibility was not affected.

**Fig 1:**
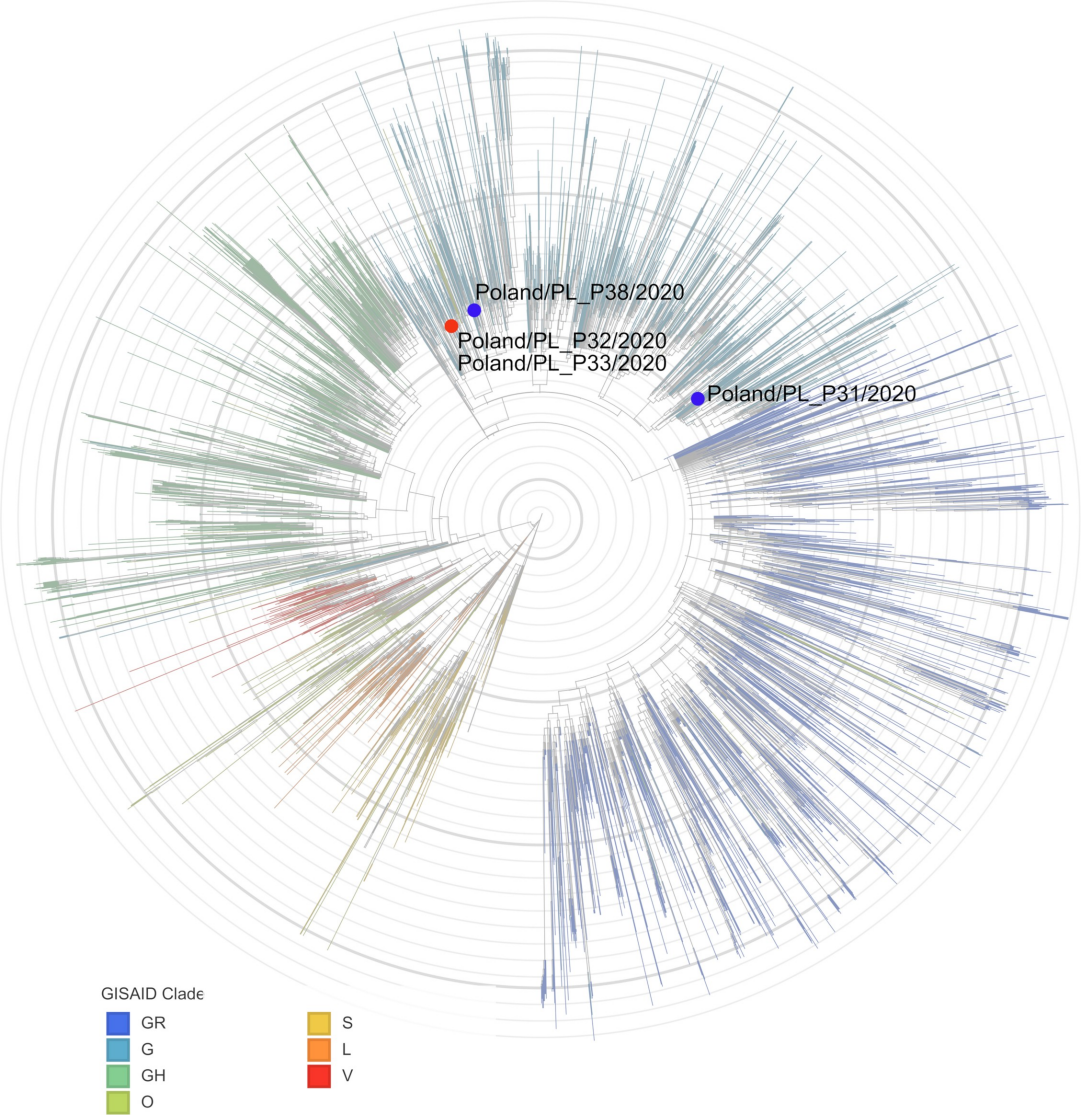
Phylogenetic analysis of the isolates included in the study. The analysis was carried out using the nexstrain server based on GISAID data [12,13], with the dataset dated on 4^th^ August 2020 [14,15]. The strains with the point mutation in the ORF10 are labeled in red, while the reference strains are labeled in blue.

To further characterize the phenotype of isolates, available clinical samples were overlaid on the fully confluent Vero E6 cells. Simultaneously, parallel cultures were inoculated with closely related PL_P31 and PL_P38 isolates (see Fig 1).

**Fig 2:**
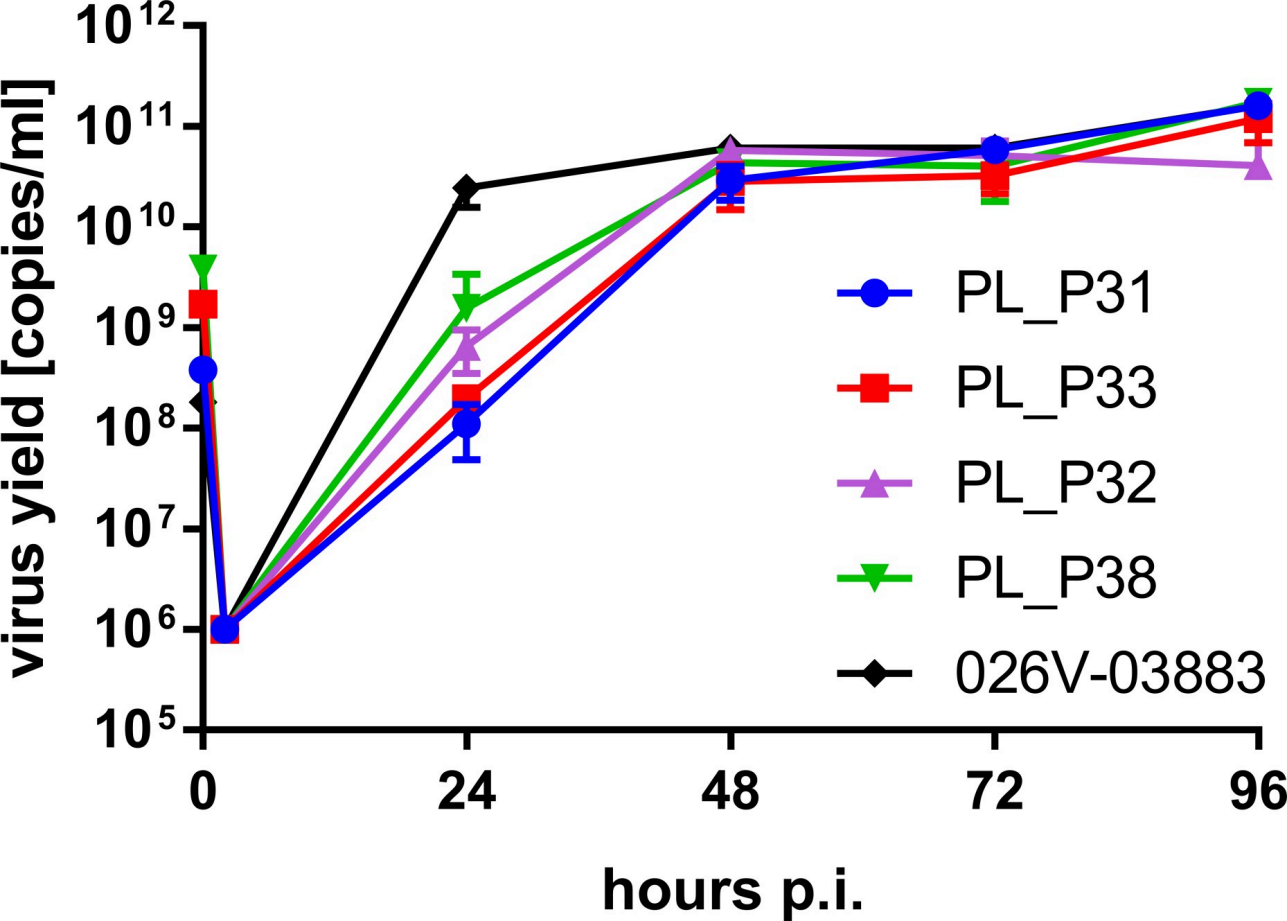
Replication kinetics of the SARS-CoV-2 strains. Virus yield was determined with RT-qPCR, and the data is presented as a mean ±SD. The EVAg strain was used as a reference.

In all four cases, 72 h post-inoculation, we observed the appearance of a characteristic CPE. The media samples were collected daily, and total RNA was isolated. The RT-qPCR reaction was carried out, and the virus yields are presented in Fig 2. No difference between the replication dynamics between strains carrying the nonsense mutation in the ORF10 and the strains with intact ORF10 was observed. The genomes of all the strains were re-sequenced after the passage, and in all the cases, the sequences were identical to the ones observed for clinical isolates.

Concluding, results obtained from the cell culture, sequencing, and clinical data show that the stop codon in the two-thirds of the protein did not affect the virus fitness. This observation further supports the thesis that the ORF10 should not be treated as the protein-coding gene, and the genome annotations should be altered [4]. This is in line with the reports from others, who could not identify the ORF10 protein and found only a marginal number of transcripts corresponding to the ORF10 [5,11]. On the other hand, ORF10 is relatively conserved, suggesting the importance of this region, e.g., due to the secondary RNA structures.

## Materials and methods

### Cells and the virus

Vero E6 (*Cercopithecus aethiops*; kidney epithelial; CRL-1586) were cultured in Dulbecco’s MEM (Thermo Fisher Scientific, Poland) supplemented with 3% fetal bovine serum (heat-inactivated; Thermo Fisher Scientific, Poland) and antibiotics: penicillin (100 U/ml), streptomycin (100 μg/ml), and ciprofloxacin (5 μg/ml). Cells were maintained at 37°C under 5% CO_2_.

The strains with the nonsense mutation in the ORF10 gene were designated names PL_P32 and PL_P33 [GISAID [12,13] Clade G, Pangolin lineage B.1] (accession numbers for the GISAID database: hCoV-19/Poland/PL_P32/2020 and hCoV-19/Poland/PL_P33/2020, respectively) and the reference samples showing high similarity on the nucleotide level, but lacking the point mutation, were designated names PL_P31 [GISAID Clade G, Pangolin lineage B.1] and PL_P38 [GISAID Clade G, Pangolin lineage B.1.5] (accession numbers for the GISAID database: hCoV-19/Poland/PL_P31/2020 and hCoV-19/Poland/PL_P38/2020). Reference SARS-CoV-2 strain 026V-03883 was kindly granted by Christian Drosten, Charité–Universitätsmedizin Berlin, Germany by the European Virus Archive—Global (EVAg); https://www.european-virus-archive.com/).

All SARS-CoV-2 stocks were generated by infecting monolayers of Vero E6 cells. The virus-containing liquid was collected at day 3 post-infection (p.i.), aliquoted, and stored at −80°C. Control samples from mock-infected cells were prepared in the same manner. Virus yield was assessed by titration on fully confluent Vero E6 cells in 96-well plates, according to the method of Reed and Muench. Plates were incubated at 37°C for three days, and the cytopathic effect (CPE) was scored by observation under an inverted microscope.

### Sequencing

Total RNA was isolated from the throat swabs collections stored as frozen PBS suspensions at -20°C using a manual TRI Reagent–chloroform extraction and sodium acetate–ethanol precipitation (Sigma-Aldrich, Poznań, Poland). The presence of SARS-CoV-2 material in the collected sample was tested using GeneFinder real-time COVID-19 plus kit (OSANG Healthcare, Korea). Isolated total RNA was treated with DNAse I to remove DNA contamination, reverse transcribed with SuperScript IV and random oligohexamer primers, next second strand synthesis was completed using DNA polymerase I (all reagents from Thermo Fisher, Warszawa, Poland). Illumina platform sequencing libraries were prepared using Nextera Flex Enrichment Library with Respiratory Virus Oligo Panel capture workflow according to the manufacturer instruction Illumina–Analitik, Warszawa, Poland). Two libraries of 12 samples barcoded with individual i7 and i5 adapters were sequenced in each run. NGS sequencing was accomplished using MiSeq v.3 2x75 chemistry (Illumina). Raw sequencing files were demultiplexed using IlluminaBasecallsToFasq procedure from PICARD package and mapped to NC_055512.2 SARS-CoV-2 reference sequence with BwaAndMarkDuplicatesPipelineSpark procedure from GATK v.4.1.5.0 package (Broad Institute, Boston, MA). Individual samples files were manually inspected using Integrated Genomics Viewer (Broad Institute). Only 2 samples out of 72 sequenced had identical C>T transition at NC_0055512:29642 position within the putative orf10 at 3’ of the virus genome. Base T read quality value was QV = 38, and the numbers of reads were 265 and 340 for samples PL_P32 and PL_P33. This transition could change putative codon 29 from glutamine (CAA, id-gu280_gp11.2) to the stop (TAA). No other sequence variants were detected in the orf10 region. Sequence alignments of samples PL_P32 and PL_P33 are in **S1** and **S2 Files**, respectively.

### Isolation of nucleic acids and reverse transcription

A viral DNA/RNA kit (A&A Biotechnology, Poland) was used for nucleic acid isolation from cell culture supernatants. RNA was isolated according to the manufacturer’s instructions. According to the manufacturer's instructions, cDNA samples were prepared with a high-capacity cDNA reverse transcription kit (Thermo Fisher Scientific, Poland).

### Quantitative PCR

Viral RNA was quantified using quantitative PCR (qPCR; CFX96 Touch real-time PCR detection system; Bio-Rad, Poland). cDNA was amplified using 1× qPCR master mix (A&A Biotechnology, Poland) in the presence of the probe (100 nM; FAM/BHQ1, ACT TCC TCA AGG AAC AAC ATT GCC A) and primers (450 nM each; CAC ATT GGC ACC CGC AAT C and GAG GAA CGA GAA GAG GCT TG). The heating scheme was as follows: 2 min at 50°C and 10 min at 92°C, followed by 30 cycles of 15 s at 92°C and 1 min at 60°C. In order to assess the copy number for the N gene, standards were prepared and serially diluted.

### *In silico* analysis of the occurrence of new premature stop codons

The relative number (by ORF size) of premature termination mutations was calculated with 42,227 high-quality SARS-CoV-2 sequences (without ambiguous nucleotides) from GISAID. The coefficient of occurrence of premature termination mutations was calculated using the number of samples with new premature mutations generating stop codons divided by the number of codons in particular ORFs and was further normalized by multiplication by a factor of 100,000/42,227, to scale the result properly for a clearer understanding of the magnitude.

## Supporting information

S1 File Sequence alignment for sample PL_P32. (ZIP)

S2 File Sequence alignment for sample PL_P33. (ZIP)

## References

[1] Fields BN, Knipe DM, Howley PM. Fields virology. 6th ed Philadelphia: Wolters Kluwer Health/Lippincott Williams & Wilkins; 2013 1 online resource (2 volumes (xx, 2456, I–82 pages)) p.

[2] Wu F, Zhao S, Yu B, Chen YM, Wang W, Song ZG, et al A new coronavirus associated with human respiratory disease in China. Nature. 2020;579(7798):265–9. Epub 2020/02/06. 10.1038/s41586-020-2008-3 32015508 PMC7094943

[3] Finkel Y, Mizrahi O, Nachshon A, Weingarten-Gabbay S, Yahalom-Ronen Y, Tamir H, et al The coding capacity of SARS-CoV-2. biorxiv repository. 2020 10.1038/s41586-020-2739-1 32906143

[4] Kim D, Lee JY, Yang JS, Kim JW, Kim VN, Chang H. The Architecture of SARS-CoV-2 Transcriptome. Cell. 2020;181(4):914–21 e10. Epub 2020/04/25. 10.1016/j.cell.2020.04.011 32330414 PMC7179501

[5] Davidson AD, Williamson MK, Lewis S, Shoemark D, Carroll MW, Heesom KJ, et al Characterisation of the transcriptome and proteome of SARS-CoV-2 reveals a cell passage induced in-frame deletion of the furin-like cleavage site from the spike glycoprotein. Genome Medicine. 2020;12(1):68 10.1186/s13073-020-00763-0 32723359 PMC7386171

[6] Alm E, Broberg EK, Connor T, Hodcroft E, Komissarov AB, Maurer-Stroh S, et al Geographic and temporal distribution of SARS-CoV-2 clades in the WHO European Region from January to June 2020. Eurosurveillance. 2020;25(32). 10.2807/1560-7917.ES.2020.25.32.2001410 PMC7427299 32794443

[7] Milewska A, Kindler E, Vkovski P, Zeglen S, Ochman M, Thiel V, et al APOBEC3-mediated restriction of RNA virus replication. Sci Rep. 2018;8(1):5960 Epub 2018/04/15. 10.1038/s41598-018-24448-2 29654310 PMC5899082

[8] Pyrc K, Jebbink MF, Berkhout B, van der Hoek L. Genome structure and transcriptional regulation of human coronavirus NL63. Virol J. 2004;1:7 Epub 2004/11/19. 10.1186/1743-422X-1-7 15548333 PMC538260

[9] Pereira F. Evolutionary dynamics of the SARS-CoV-2 ORF8 accessory gene. Infection, Genetics and Evolution. 2020;85:104525 10.1016/j.meegid.2020.104525 32890763 PMC7467077

[10] Corman VM, Landt O, Kaiser M, Molenkamp R, Meijer A, Chu DK, et al Detection of 2019 novel coronavirus (2019-nCoV) by real-time RT-PCR. Euro Surveill. 2020;25(3). Epub 2020/01/30. 10.2807/1560-7917.Es.2020.25.3.2000045 31992387 PMC6988269

[11] Bojkova D, Klann K, Koch B, Widera M, Krause D, Ciesek S, et al Proteomics of SARS-CoV-2-infected host cells reveals therapy targets. Nature. 2020;583(7816):469–72. 10.1038/s41586-020-2332-7 32408336 PMC7616921

[12] Elbe S, Buckland-Merrett G. Data, disease and diplomacy: GISAID's innovative contribution to global health. Global Challenges. 2017;1(1):33–46. 10.1002/gch2.1018 31565258 PMC6607375

[13] Shu Y, McCauley J. GISAID: Global initiative on sharing all influenza data–from vision to reality. Eurosurveillance. 2017;22(13):30494 10.2807/1560-7917.ES.2017.22.13.30494 28382917 PMC5388101

[14] Hadfield J, Megill C, Bell SM, Huddleston J, Potter B, Callender C, et al Nextstrain: real-time tracking of pathogen evolution. Bioinformatics. 2018;34(23):4121–3. 10.1093/bioinformatics/bty407 29790939 PMC6247931

[15] Sagulenko P, Puller V, Neher RA. TreeTime: Maximum-likelihood phylodynamic analysis. Virus Evol. 2018;4(1):vex042 Epub 2018/01/18. 10.1093/ve/vex042 29340210 PMC5758920

